# Salivary and Urinary Total Antioxidant Capacity as Biomarkers of Oxidative Stress in Humans

**DOI:** 10.1155/2016/5480267

**Published:** 2016-02-07

**Authors:** Ilaria Peluso, Anna Raguzzini

**Affiliations:** Center of Nutrition, Council for Agricultural Research and Economics (CREA-NUT), Via Ardeatina 546, 00178 Rome, Italy

## Abstract

Total Antioxidant Capacity (TAC) is a biomarker often used in order to investigate oxidative stress in many pathological conditions. Saliva and urine can be collected noninvasively and represent attractive diagnostic fluids for detecting biomarkers of various pathological conditions. The reviewed case-control and intervention studies that measured salivary or urinary TAC revealed that diseases, antioxidant foods, or supplements and age, gender, and lifestyle factors influenced salivary or urinary TAC. Salivary and urinary TAC were particularly affected by oral or renal status, respectively, as well as by infection; therefore these factors must be taken into account in both case-control and intervention studies. Furthermore, some considerations on sample collection and normalization strategies could be made. In particular, unstimulated saliva could be the better approach to measure salivary TAC, whereas 24 h or spontaneous urine collection should be chosen on the basis of the study outcome and of the creatinine clearance. Finally, the uric acid-independent TAC could be the better approach to evaluate red-ox status of body, in particular after nutritional interventions and in diseases associated with hyperuricaemia.

## 1. Introduction

Oxidative stress, defined as the imbalance between Reactive Oxygen Species (ROS) production and antioxidant defense inside human organism, is a risk factor playing a significant pathogenetic role for noncommunicable diseases [1]. A nonphysiological ROS production originates either by xenobiotics or by endogenous sources [2], such as the respiratory burst [3]. Synergistic interactions between antioxidants, in part involving antioxidant regeneration, must be taken into account in order to properly assess antioxidant status in vivo. Total Antioxidant Capacity (TAC), defined as the moles of oxidants neutralized by one litre of solution, is a biomarker measuring the antioxidant potential of body fluids [4]. Several reviews described the various assays commonly used for the measurement of TAC [5–8] and a good correlation between the results obtained with different methods was found [9, 10]. Salivary and urinary collections are simple and noninvasive and this is the reason why TAC of saliva or urine has led to increasing interest. It has been suggested that saliva could constitute a first line of defense against free radical-mediated oxidative stress [11], whereas the composition of urine reflects the continuously changing environment of the body, which is affected, among other factors, by diet and in particular by polyphenol metabolites excreted in urine [12].

We aimed to review case-control and intervention studies that measured salivary [13–138] or urinary [138–179] TAC.

## 2. Infection and Oral and Renal Status

Saliva and urine are particularly affected by oral or renal status, respectively, as well as by infection; therefore the relationships of salivary and urinary TAC with these conditions have been investigated in many studies (Table 1). Urinary TAC increased during urinary tract infection [148, 149], while decreased levels of salivary TAC were observed during* H. pylori* [130] and HIV [98, 112] infections and after a single consumption of enterococci containing Bryndza cheese [63]. The latter temporally affected the composition of oral microbiota [63].

On the contrary, dental caries, one of the most common infectious diseases worldwide, was associated with higher salivary TAC (Table 1). Therefore, the relationship between infection and TAC depends on the type and on the site of infection.

On the other hand, oxidative stress is implicated in the pathogenesis of many oral diseases and meta-analysis results showed that TAC levels from peripheral blood samples were significantly different between periodontitis (PD) patients and healthy subjects, suggesting that chronic periodontitis is associated with systemic oxidative stress in human bodies [180]. In agreement with this finding, lower values of salivary TAC were found in PD patients versus healthy subjects in 66.7% of the studies (Table 1). When increased salivary TAC levels were observed, it has been suggested that this increase may represent an adaptive response to oxidative stress in PD [115]. Dental hygiene procedures and scaling and root planing increased salivary TAC in 100% of the studies conducted on healthy subjects with caries and in 50% of the studies on patients with PD (Table 1). Therefore, the antioxidant status in saliva is related to both oral hygiene and periodontal status.

On the other hand, 66.7% of the studies that have investigated the salivary TAC in more serious oral diseases, including recurrent aphthous stomatitis (RAS) (the most common oral ulcerative condition) [36, 88], stem cell transplantation- (SCT-) related salivary gland injury due to graft versus host disease (GVHD) [92], oral mucositis induced by high-dose therapy of melphalan in myeloma patients treated with autologous SCT [22], cleft lip and palate [17], oral premalignant lesions (leukoplakia, lichen planus, and erythroplakia) [13–15, 23, 29, 49, 83, 113, 128], and oral cancers [14, 24], found decreased levels of salivary TAC in these patients (Table 1). Furthermore, salivary TAC was decreased also in patients with peri-implant disease [81] and in children undergoing fixed orthodontic therapy [54].

Concerning renal status, decreased levels of urinary TAC have been reported in renal diseases including secondary (induced by hypertension and diabetes) and primary renal disease, chronic and acute renal failure, and shockwave lithotripsy- (SWL-) induced acute kidney injury (Table 1).

Decreased urinary TAC has been reported 120 minutes after SWL in both patients with kidneys stone and controls [158], but others reported that SWL did not change urinary TAC measured in the 24-h urine [179]. On the contrary, urinary TAC was higher in childhood urolithiasis and was associated with hyperuricosuria [154].

Chronic renal failure is often treated with peritoneal dialysis and chronic renal failure patients undergoing dialysis are characterized by decreased urinary TAC [142]. However, both increased and decreased salivary TAC was found in these patients [30, 31, 86] (Table 1), probably due to the hyperuricaemia. Kidney transplantation is the treatment of choice for most patients with end-stage renal disease and it has been suggested that urinary biomarkers of oxidative stress could be useful in the assessment of kidney quality before transplantation, which is needed to predict recipient outcomes and to optimize management and allocation of the allograft [181]. In particular, TAC was found to be significantly lower in donor urine for kidneys from poor cadaveric donors, based on clinical impression or renal transplant outcome, compared to urinary TAC of living related donors and good cadaveric donors [173, 174]. On the other hand, increased urinary TAC was found in patients with delayed graft function, but not with early graft function [174].

## 3. Systemic Diseases

Oxidative stress is associated with the metabolic syndrome, a cluster of cardiovascular risk factors including dyslipidemia, abnormal glucose tolerance, hypertension, and obesity [182]. Despite the antioxidant effect of uric acid (UA), hyperuricaemia is associated with obesity and insulin resistance [183] and has been proposed as a component of the metabolic syndrome [184, 185].

Contrasting results came from the studies that investigated salivary TAC in subjects with at least one of the metabolic syndrome symptoms (Table 2).

Pregnant women with diabetes were found to have increased salivary TAC, but also markedly increased indexes of caries activity [119]. Besides the authors [119] found increased* Lactobacillus* and* Streptococcus* species in saliva of pregnant women with diabetes having systemic complications as compared to healthy individuals and other patients. However, increase in salivary TAC was observed also in obese subjects with hyperuricaemia [50] and in obese children despite their good oral hygiene status [56]. While rasburicase infusion decreased salivary TAC [50], intravenous N-acetylcysteine failed to prevent renal dysfunction and oxidative stress after contrast media administration during percutaneous coronary interventions [144] (Table 2). On the other hand, only hypertensive patients in treatment with metoprolol had lower salivary TAC compared to controls [46] (Table 2).

Salivary or urinary TAC was studied also in other diseases, such as cancer, neuropsychiatric disorders, congenital and genetic diseases, and immune mediated and inflammatory diseases (Table 2).

Urinary TAC was decreased in bladder cancer [165] and in nonsmokers with lung cancer [152], but not in smokers with lung cancer. Decreased salivary TAC was observed in patients with squamous cell carcinoma of head and neck [74] and with brain tumor [118], but increased salivary TAC was reported in acute lymphoblastic leukemia (ALL) children [59] (Table 2). Only children with more than 2 weeks of chemotherapy had decreased levels of salivary TAC [59].

Increased levels of salivary TAC were found in patients with neurological disorders and tube-feeding [42], whereas decreased levels were reported in cerebral palsy [117], intractable epilepsy [111], and autistic children [105]. Also urinary TAC levels were lower in autistic compared to healthy children [150, 167].

On the other hand, case-control studies on congenital or genetic diseases such as Down syndrome [43, 116] and cystic fibrosis [82] reported conflicting results on salivary TAC (Table 2). Besides, Campos et al. [146] found increased and decreased levels of urinary TAC in children and adults with Down syndrome, respectively (Table 2). On the contrary, urinary TAC resulted in a good marker of oxidative stress in patients with inborn errors of metabolism (IEM), such as maple syrup urine disease (MSUD), propionic aciduria (PA), methylmalonic aciduria (MMA), 3-hydroxy-3-methylglutaric aciduria (HMGA), and ornithine transcarbamylase deficiency (OTC), both before and after treatment (Table 2).

On the other hand, salivary TAC was lower in asthma [58], Crohn's disease [106], multiple sclerosis [65, 135], and Sjögren's syndrome [109] patients compared to healthy individuals (Table 2), but neither corticosteroid therapy, in multiple sclerosis patients [65], nor continuous positive airway pressure (CPAP) in obstructive sleep apnea syndrome (OSAS) improved salivary TAC [38, 123]. Furthermore, increased salivary TAC levels were found in chronic obstructive pulmonary disease (COPD) [132] and in juvenile idiopathic arthritis (JIA) [33]. Children with JIA, whether treated or not treated with anti-tumor necrosis factor- (TNF-) *α* agents (Infliximab or Etanercept), had higher salivary TAC and the increase of the active patients was nearly two times higher than that of nonactive patients [33]. Increased salivary TAC levels were found also in pre- and postsurgical patients, where surgical procedures, involving stress such as extended general anaesthesia and a long presurgical fasting period, may cause systemic inflammation and oxidative stress [126]. Increased salivary TAC was also observed in Regional Pain Syndrome type I [48], whereas decreased salivary TAC levels were reported in temporomandibular disorders [108].

## 4. Nutritional and Supplement Interventions

Although body possesses a sophisticated and cooperative array of endogenous antioxidant defenses, dietary consumption of antioxidant-rich foods may lower the risk of noncommunicable diseases, by increasing TAC. In fact, 70% of the interventions with plant foods or supplements conducted on subjects characterized by oxidative stress conditions such as cardiovascular risk factors (smoking, hypercholesterolemia, metabolic syndrome, hyperlipidemia, and hypertension) or with pathologies reported an increase of plasma/serum TAC [186]. In the studies that investigated the effect of dietary or supplementation interventions on salivary or urinary TAC (Table 3), volunteers were prevalently healthy; however, in some studies, overweight (OW) [164] or elderly [94, 143, 147, 153] subjects, smokers [25], or patients with PD [137] or dialyzed [142] were enrolled. A review of 41 interventions, from 29 studies, has been performed (Table 3). Of these interventions, 18 regard a single ingestion (bolus) and the other 23 were related to repeated supplementations.

Caffeinated or alcoholic antioxidant beverages increased salivary TAC in 50% and urinary TAC in 40% of the interventions. In particular, green tea (GT) consumption for 4 or 12 weeks increased salivary TAC in laboratory workers and elderly subjects [94, 120], whereas it did not affect salivary TAC of Taekwondo (TKD) athletes after training [80]. Besides, Benzie et al. [141] found a significant correlation between urinary FRAP values and urinary total phenolic concentrations after GT consumption. Red wine bolus consumption did not change salivary TAC [127] but increased urinary TAC in elderly women [147]. After a single or 2 weeks of consumption of green or black coffee urinary TAC did not change [138, 168].

On the other hand, walnuts [160, 166] increased urinary TAC both after 4 weeks and after a single consumption and cocoa increased urinary TAC at 6–12 h after intake concomitantly with the excretion of epicatechin urinary metabolites [170] (Table 3).

Concerning fruit, juices, and vegetables, only Zare Javid et al. [137] investigated the effect of fruits, vegetables, and whole grains (3 and 6 months) consumption on salivary TAC of PD patients and did not find any change in this marker. Only one study reported decreased urinary TAC levels after a single consumption of blackberry juices [157]. On the contrary, urinary TAC increased after bolus consumption of spinach [147], strawberries [147], Jerte Valley cherry [153], and a fruit based drink (86% of a mix of apple, grape, blueberry and pomegranate juices and grape skin, grape seed, and green tea extracts) ingested during a high fat meal (HFM) [164]. In the same postprandial study, a less antioxidant fruit based drink (63% of a mix of pineapple, black currant, and plum juices) during HFM did not affect urinary TAC, a well as plasma TAC [164]. Also grape juice consumption for 5 days increased urinary TAC [155], whereas tomato juice consumption for 2 weeks did not [161]. The same study found increased urinary TAC levels when tomato juice was supplemented with vitamin C (870 mg/L) fortification [161]. Vitamin C (250 mg) with vitamin E (400 i.u.) supplementation for 8 weeks increased urinary TAC in dialyzed patients [142] and vitamin C in bolus administration increased urinary TAC in elderly women (1250 mg) [147] and salivary TAC in healthy subjects (250 mg) [64]. On the contrary neither vitamin C after 3 weeks (500 mg) of consumption in smokers [25] nor 90 days of astaxanthin supplementation in trained male soccer players in preexercise conditions [27] changed salivary TAC. Conversely, red wine extract administration caused a marked rise in salivary TAC, within 30 min, and the same treatment raised also salivary polyphenol concentration [127]. Helbig et al. [159] reported the same increase in urinary TAC versus baseline with both control bread and bread enriched with black currant press residue (4 weeks). Besides, urinary TAC was unchanged after 21 days consumption of a dried fruit and vegetable extracts fortified with antioxidants [176], after 2 weeks of Pycnogenol (200 mg/day) supplementation [175] and after 24 days of administration of an antioxidant mixture containing vitamin E, beta-carotene, ascorbic acid, selenium, alpha-lipoic acid, N-acetyl 1-cysteine, catechin, lutein, and lycopene [171]. On the contrary, ganoderma lucidum (Lingzhi, woody mushroom) supplements increased urinary TAC both in bolus and in repeated administration [177, 178].

Concerning other dietary interventions, a low fat diet did not affect salivary TAC compared to a high fat diet [32] and a HFM did not affect urinary TAC [164]. On the contrary, a fish diet (8 oz/week of salmon) for 4 weeks [160] and the consumption of tryptophan-enriched cereals for 1 week [143] increased urinary TAC.

## 5. Age, Gender, and lifestyle Factors

Age and gender differences were observed in oxidative stress markers in plasma [187]. It was found that salivary TAC increased with the age of the children with caries [60], as well as during aging [62, 69]. On the contrary, others reported that salivary TAC was higher in younger subjects [73] and negative correlations with age were found for urinary TAC [146]. Women had significantly lower salivary TAC than men [79, 110, 114]. In this context Kawamoto et al. [66] reported decreased levels of salivary TAC during the ovulatory phase compared to follicular phase in women with PD, but not in healthy women. TAC was negatively correlated with bacterial counts during the ovulatory phase, but not during the follicular phase [66]. Besides, salivary TAC was lower during uncomplicated pregnancy [70] and in osteoporotic subjects compared to age-matched healthy women [133].

Apart from age, gender, diseases, and dietary habit, also lifestyle factors could affect salivary and urinary TAC. Within lifestyle factors, many studies investigated the effect of exercise and smoking habit on salivary or urinary TAC (Table 4).

In agreement with the well-known reduction of antioxidant defenses after physical stress, strong correlation was found between the variations of salivary and plasma TAC during the training season in triathletes [134]. Both 2 hr TKD training session and 1 hr exhaustive aerobic dance exercise decreased salivary TAC [21, 80]. Besides also urinary TAC decreased after 3 hours or more of TKD training camp/d for 5–8 days [140]. Only a study found increased salivary TAC in older adults with PD who performed Tai Chi during a period of 6 months; however this increase was accompanied by a statistically significant decrease in the Periodontal Disease Index (PDI) [85].

More conflicting are results on smoking habit; higher, lower, or nonsignificant different levels of salivary TAC have been found in smokers compared to nonsmokers, whereas no significant differences were found in urinary TAC between smokers and nonsmokers (Table 4).

On the contrary, alcohol-dependent subjects showed significantly lower TAC in blood and saliva as compared to those in the controls and the alcohol withdrawal caused an increase in the TAC to near-control values [102].

Decreased levels of salivary TAC were found also in workers subjected to occupational exposure to nonferrous metal mine conditions [51], whereas exposure to trichloroethylene increased urinary TAC [139].

Although there was no statistically significant effect of cell phone talking time on the salivary TAC, the latter progressively increased with time and reached maximum at 30 min, probably due to exposure to radio frequency radiation [67]. However, both increased [57] and decreased [18] levels of salivary TAC in mobile phone users were found.

On the other hand, it has been observed that salivary TAC was affected by emotional and psychological factors [20]. In particular, watching a cheerful comical video for 30 min increased salivary TAC [20].

## 6. Methodological Issues

**Figure 1:**
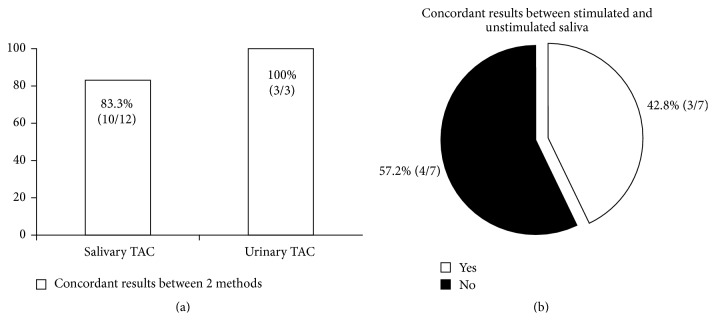
Concordat results between two different methods used in the same study to measure salivary or urinary TAC (a). Concordant results between unstimulated and stimulated saliva samples analyzed in the same study (b). References [22, 24, 34, 38, 50, 62, 64, 65, 67, 72, 78, 81, 111, 127, 135, 137, 161, 166, 175, 188–190].

Despite the differences between the assays [8], concordant results were found in the majority of the cases between the results obtained when two different methods (Trolox Equivalent Antioxidant Capacity (TEAC), Ferric Reducing Antioxidant Potential (FRAP), Oxygen Radical Antioxidant Capacity (ORAC), Total-radical Trapping Antioxidant Parameter (TRAP), or enzyme-linked immunosorbent assay (ELISA) colorimetric kit) were used in the same study to measure salivary or urinary TAC (Figure 1(a)).

In particular, although FRAP was significantly lower in multiple sclerosis patients, whereas no significant difference in salivary TEAC between patients and controls was observed [65], the authors pointed out that their study had some limitations, such as the low number of patients included, the variability of the clinical status, and the different treatment of patients. Therefore, method choice does not seem to be a critical point for salivary TAC measure. On the contrary, major concerns came from saliva collection (Figure 1(b)).

Saliva sampling has been improved by cotton and polypropylene Salivette collection systems, but the latter altered the determination of some markers [188]. It has been reported that in samples collected using the cotton Salivette TAC was comparable, but higher thiobarbituric acid reacting substances (TBARS) concentrations were determined compared with unstimulated saliva [188].

In healthy subjects values were higher under stimulated conditions for saliva flow and were higher under unstimulated conditions with respect to TAC and UA [189]. When TAC was examined with respect to flow rate, a significantly lower rate of antioxidant production was noted in patients with PD compared with controls for unstimulated saliva but not for stimulated saliva [34]. Besides, a significant decrease in UA and TAC was observed in unstimulated saliva as well as a significant increase in all antioxidants examined in stimulated saliva of systemic sclerosis women with normal salivary flow rate as compared to the healthy controls [135].

Nagler et al. [190] examined the correlation between the levels of various salivary antioxidant components and the TAC in whole saliva in comparison with those in saliva secreted specifically from the major salivary glands: the parotid and the submandibular/sublingual (Sm_Sl) glands. While the secretory IgA and lysozyme concentrations were similar in parotid and Sm_Sl saliva, the authors [190] found higher levels of the various antioxidant parameters (antioxidant enzymes, UA and TAC) in the parotid saliva as compared with the Sm_Sl saliva, especially under resting condition. Under resting condition parotid saliva was the major source of salivary antioxidants, especially of UA [190]. After stimulation (i.e., eating), the various parameters of antioxidants of parotid saliva were considerably reduced, in light of the well-reported “dilution effect” that follows stimulation. In addition, fluids by nonglandular sources contributed to the possible dilution of the whole saliva, after meal. Therefore, unstimulated saliva with and without flow rate normalization could be the better approaches to measure salivary TAC.

Also urine can be more or less diluted, complicating the evaluation of parameters in spontaneous urine. Particularly after physical stress [140] more concentrated urine is to be expected due to sweating. In order to obviate this problem and provided that the creatinine excretion is relatively constant over 24 h, the mean creatinine excretion can serve as a reference for the measured values of excretion. Generally, an average creatinine excretion of 1 g/24 h is assumed [140]. However, it must be taken into consideration that age, sex, muscle mass, and diet all have an influence on creatinine excretion [191]. Although analyzing 24 h urine collection would have been preferable in case-control studies, it was logistically impossible in some cases. On the other hand, 24 h urine collection could not appreciate the increase in TAC due to intervention with polyphenol-rich foods, considering the rate of elimination of polyphenols [12]. Therefore, 24 h or spontaneous urine collection should be chosen on the basis of the study outcome and of the creatinine clearance.

## 7. Uric Acid-Independent TAC versus TAC

**Figure 2:**
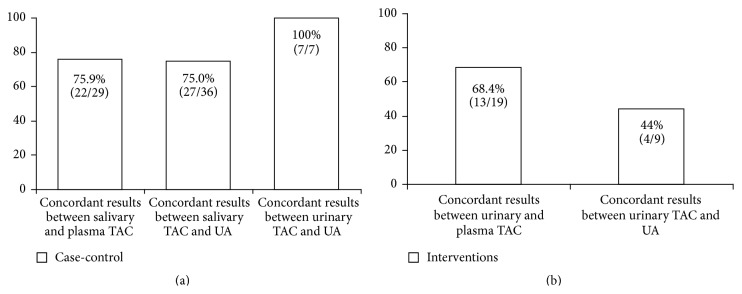
Concordant results between salivary or urinary TAC and UA levels or plasma TAC in case-control (a) or antioxidant/nutritional intervention (b) studies. References [17, 19, 21–24, 26, 28–31, 34, 37, 38, 40–44, 48–51, 55, 57, 61, 62, 65, 71, 77, 78, 81, 82, 86–89, 91, 92, 95–97, 102, 106–111, 119, 126, 132–136, 140–142, 144, 146, 147, 150, 154, 157, 159–162, 164, 166, 171, 175–178].

UA is a powerful scavenger of free radicals and provides 60–80% of TAC in plasma [192, 193]. It has been also reported that TAC of both plasma and urine is mainly related to the UA concentration of the samples [194]. Besides, UA contributes approximately 70% of the salivary TAC [89], with the antioxidant role of the ascorbic acid being secondary [89] and correlation between concentrations of UA in both saliva and plasma points to the latter as the origin of salivary UA [71, 190]. According to this hypothesis, when salivary or urinary TAC, UA, and plasma or serum TAC were measured in the same study, it turned out that there was a clear accordance between UA concentration and TAC, as well as between salivary or urinary TAC and plasma or serum TAC in case-control studies (Figure 2(a)). Furthermore, rasburicase i.v. infusion in obese subjects with hyperuricaemia caused a marked decrease in both plasma and saliva TAC values [50].

Increase in serum UA levels can be due to increased intake of dietary purines, alcohol and fructose [195], impaired renal function and renal microvascular disease, which can increase UA production and/or decrease UA clearance [196], and hyperinsulinemia, which increases renal UA reabsorption [197]. In particular, the plasma level of UA is regulated by renal function [162]. However, although urine from renal disease patients had more proteins and UA compared to controls, urinary TAC from acute renal failure patients did not correlate with UA [162]. Furthermore, contrasting results came from patients with severe chronic renal failure (CRF) (dialytic) without diabetes and severe CRF (dialytic) with diabetes [30]. In severe-CRF patients without diabetes, median TAC and UA levels decreased following dialysis, whereas in severe-CRF patients with diabetes, median TAC increased following dialysis while median UA decreased [30].

Although UA in urine samples constitutes about 75% of urinary TAC [140], the composition of urine is unpredictable, reflecting the continuously changing environment of the body, which is affected, among other factors, by diet [162]. In fact, only in 44% (4/9) of the interventions with antioxidant foods, beverages or supplements urinary TAC was related to UA (Figure 1(b)). These findings are probably due to the fact that low molecular weight antioxidants, such as polyphenols, are cleared from the blood by kidney [12]. The only study that [137] investigated the effect of fruits, vegetables, and whole grains consumption on both salivary and plasma TAC did not find any change in salivary TAC, despite the increase in plasma TAC.

In order to avoid the UA interference, methods for UA-independent TAC have been proposed, by using the uricase reaction, in both plasma and urine [198], or by using the corrected TAC, the calculated parameter that represents the fraction of circulating antioxidants after the elimination of the interference of UA [140, 199].

Similar calculated urinary TAC has been proposed by Campos et al. [146]. Authors suggested that TAC-UA/Cr could provide more reliable information about the antioxidant status, at least in Down syndrome, because children had higher TAC/Cr and UA/Cr, whereas levels of TAC-UA/Cr of adult patients were lower compared to healthy subjects.

On the contrary, uricase methods could be biased by the generation of H_2_O_2_ during the action of uricase [200]. Therefore, the calculated corrected TAC-UA could be the better approach.

## 8. Conclusion

The reviewed case-control and intervention studies that measured salivary or urinary TAC revealed that these markers can be useful in the evaluation of the antioxidant status of the body taking into account some factors. First of all, salivary and urinary TAC were particularly affected by oral or renal status, respectively, as well as by infection (Table 1); therefore these factors could bias results in some studies [66, 85, 119]. Second, samples collection (Figure 1) and normalization strategies should be chosen on the basis of the type of biological fluid, of the study outcome, and of the creatinine clearance. Again, TAC is related to both UA (Figure 2) and nutritional antioxidant levels in biological fluids [127, 141, 147, 170]. From that, the UA-independent TAC could be the better approach to evaluate red-ox status of body both after nutritional interventions and in diseases associated with hyperuricaemia. Finally, TAC is sensitive also to the endogenous antioxidant response induced by some pathological and environmental conditions [20, 33, 48, 57, 59, 67, 115, 126, 132, 139, 142].

## Figures and Tables

**Table 1:** Infection and oral and renal status.

References	Case-control studies	Salivary TAC percentage (*n*°)	Urinary TAC percentage (*n*°)	Treatment effect percentage (*n*°)
[98, 112, 130, 148, 149]	Infection	↓ 100% (3/3)	↑ (100%, 2/2)	Salivary TAC ↑ HAART in HIV+

[16, 45, 47, 60, 61, 64, 72, 73, 75, 78, 84, 90, 99, 103, 104, 122, 124, 125]	Caries	↑ 81.2% (13/16) *↔* 6.3% (1/16) ↓ 12.5% (2/16)		Dental hygiene procedures ↑ 100% (3/3)

[26, 28, 34, 35, 37, 39, 40, 44, 52, 53, 66, 68, 76, 87, 89, 95, 96, 100, 101, 110, 114, 115]	Periodontitis	↓ 66.7% (14/21) ↔ 23.8% (5/21) ↑ 9.5% (2/21)		Dental hygiene procedures or scaling and root planing ↑ 50% (3/6) ↔ 50% (3/6)

[13–15, 17, 22–24, 29, 36, 49, 83, 88, 92, 113, 128]	Severe oral diseases	↓ 66.7% (10/15) ↔ 26.7% (4/15) ↑ 6.6% (1/15)		

[30, 31, 86, 142, 154, 158, 162, 179]	Renal diseases	↑ 50% (2/4) ↓ 50% (2/4)	↓ 60% (3/5) ↔ 20% (1/5) ↑ 20% (1/5)	

HAART: highly active antiretroviral therapy; TAC: total antioxidant capacity; ↑: increase; ↓: decrease; ↔: unchanged.

**Table 2:** Systemic diseases.

References	Case-control studies	Salivary TAC percentage (*n*°)	Urinary TAC percentage (*n*°)	Treatment effect percentage (*n*°)
[19, 37, 46, 50, 55, 56, 91, 100, 101, 107, 119, 129, 144]	Metabolic syndrome symptoms	↑ 41.7% (5/12) ↔ 33.3% (4/12) ↓ 25% (3/12)		Salivary TAC ↔ 100% (1/1) enalapril ↓ 100% (1/1) metoprolol ↓ 100% (1/1) rasburicase Urinary TAC ↓ 100% (1/1) PCI ↔ 100% (1/1) NAC before and after PCI

[59, 74, 118, 152, 165]	Cancer	↓ 66.7% (2/3) ↑ 33.3% (1/3)	↓ 66.7% (2/3) ↔ 33.3% (1/3)	Salivary TAC ↓ chemotherapy

[42, 105, 111, 117, 150, 167]	Neuropsychiatric disorders	↓ 75% (3/4) ↑ 25% (1/4)	↓ 100% (2/2)	

[43, 82, 116, 146, 151, 156, 163, 169]	Congenital and genetic diseases	↓ 33.3% (1/3) ↔ 33.3% (1/3) ↑ 33.3% (1/3)	↓ 83.3% (5/6) ↑ 16.7% (1/6)	Urinary TAC: ↑ 100% (3/3) low-protein diet + L-carnitine in IEM patients

[33, 38, 48, 58, 65, 106, 108, 109, 123, 126, 132, 135]	Immune mediated and inflammatory diseases	↓ 60% (6/10) ↑ 40% (4/10)		Salivary TAC ↔ 100% (1/1) corticosteroid ↔ 100% (2/2) CPAP in OSAS ↔ 100% (1/1) anti-TNF*α* therapy

CPAP: continuous positive airway pressure; IEM: inborn errors of metabolism; NAC: N-acetylcysteine; OSAS: obstructive sleep apnea syndrome; PCI: percutaneous coronary intervention; TAC: total antioxidant capacity; TNF*α*: tumor necrosis factor alpha; ↑: increase; ↓: decrease; ↔: unchanged.

**Table 3:** Nutritional and supplement interventions.

References	Intervention	Number of bolus/repeated consumptions	Salivary TAC percentage (*n*°)	Urinary TAC percentage (*n*°)
[80, 94, 120, 127, 138, 141, 147, 168]	Caffeinated or alcoholic antioxidant beverages	5/4	↑ 50% (2/4) ↔ 50% (2/4)	↑ 40% (2/5) ↔ 60% (3/5)

[160, 166, 170]	Cocoa powder and walnuts	2/1		↑ 100% (3/3)

[137, 147, 153, 155, 157, 161, 164]	Fruit, juices, and vegetables	6/3	↔ 100% (1/1)	↑ 62.5% (5/8) ↓ 12.5% (1/8) ↔ 25% (2/8)

[25, 27, 64, 127, 142, 147, 159, 161, 171, 175–178]	Supplements and supplemented foods	4/12	↑ 50% (2/4) ↔ 50% (2/4)	↑ 58.3% (7/12) ↔ 41.7% (5/12)

[32, 143, 160, 164]	Other dietary interventions	1/3	*↔* 100% (1/1)	↑ 66.7% (2/3) ↔ 33.3% (1/3)

TAC: Total Antioxidant Capacity.

**Table 4:** Life-style factors.

References	Factor	Salivary TAC percentage (*n*°)	Urinary TAC percentage (*n*°)
[21, 80, 85, 134, 140]	Exercise	↓ 75% (3/4) ↑ 25% (1/4)	↓ 100% (1/1)

[32, 35, 41, 53, 71, 77, 93, 132, 136, 138, 145]	Smoking habit	↑ 20% (2/10) ↔ 50% (5/10) ↓ 30% (3/10)	↔ 100% (1/1)

[102]	Alcohol dependence	↓ 100% (1/1)	

[51, 139]	Occupational exposure to toxicants	↓ 100% (1/1)	↑ 100% (1/1)

[18, 20, 57, 67]	Cell phone and watching TV	↑ 75% (3/4) ↓ 25% (1/4)	

TAC: Total Antioxidant Capacity.
